# 3D airway geometry analysis of factors in airway navigation failure for lung nodules

**DOI:** 10.1186/s40644-024-00730-7

**Published:** 2024-07-04

**Authors:** Hwan-ho Cho, Junsu Choe, Jonghoon Kim, Yoo Jin Oh, Hyunjin Park, Kyungjong Lee, Ho Yun Lee

**Affiliations:** 1 Department of Electronics Engineering, Incheon National University, Incheon, Republic of Korea; 2 Division of Pulmonary and Critical Care Medicine, Department of Medicine, Samsung Medical Center, Sungkyunkwan University School of Medicine, 81 Irwon-ro, Gangnam-gu, Seoul, 06351 Republic of Korea; 3 Department of Health Sciences and Technology, SAIHST, Sungkyunkwan University, Seoul, 06351 South Korea; 4 Department of Radiology and Center for Imaging Science, Samsung Medical Center, Sungkyunkwan University School of Medicine, 81 Irwon-Ro, Gangnam-Gu, Seoul, 06351 Korea; 5 Department of Electronic and Computer Engineering, Sungkyunkwan University, Suwon, Korea; 6 Center for Neuroscience Imaging Research, Institute for Basic Science, Suwon, Republic of Korea

**Keywords:** Bronchoscopy, Solitary pulmonary nodule, Tomography scanners, X-Ray computed

## Abstract

**Background:**

This study aimed to quantitatively reveal contributing factors to airway navigation failure during radial probe endobronchial ultrasound (R-EBUS) by using geometric analysis in a three-dimensional (3D) space and to investigate the clinical feasibility of prediction models for airway navigation failure.

**Methods:**

We retrospectively reviewed patients who underwent R-EBUS between January 2017 and December 2018. Geometric quantification was analyzed using in-house software built with open-source python libraries including the Vascular Modeling Toolkit (http://www.vmtk.org), simple insight toolkit (https://sitk.org), and sci-kit image (https://scikit-image.org). We used a machine learning-based approach to explore the utility of these significant factors.

**Results:**

Of the 491 patients who were eligible for analysis (mean age, 65 years +/- 11 [standard deviation]; 274 men), the target lesion was reached in 434 and was not reached in 57. Twenty-seven patients in the failure group were matched with 27 patients in the success group based on propensity scores. Bifurcation angle at the target branch, the least diameter of the last section, and the curvature of the last section are the most significant and stable factors for airway navigation failure. The support vector machine can predict airway navigation failure with an average area under the curve of 0.803.

**Conclusions:**

Geometric analysis in 3D space revealed that a large bifurcation angle and a narrow and tortuous structure of the closest bronchus from the lesion are associated with airway navigation failure during R-EBUS. The models developed using quantitative computer tomography scan imaging show the potential to predict airway navigation failure.

**Supplementary Information:**

The online version contains supplementary material available at 10.1186/s40644-024-00730-7.

## Background

Lung cancer is the leading cause of cancer-related death worldwide, and early diagnosis is crucial for survival [1]. Although imaging modalities have advanced for decades, pathology remains essential in lung cancer treatment decisions. For the diagnosis of peripheral lung nodules, percutaneous needle aspiration has been the preferred procedure for obtaining lung tissue. However, recent advances in bronchoscopy are leading to the development of cutting-edge techniques, including radial endobronchial ultrasound (R-EBUS), electromagnetic navigation bronchoscopy, and robotic bronchoscopy. The most widely used of these, R-EBUS, had a pooled sensitivity as low as 0.72 in a recent meta-analysis investigating 7,601 patients [2].

R-EBUS is unable to reach 7–28% of peripheral pulmonary nodules [3]. Airway navigation failure is defined as the inability to access the target lesion during navigation bronchoscopy, resulting in the inability to obtain tissue from lung nodules. Predicting airway navigation failure before a bronchoscopy can reduce unnecessary procedures. Although the presence of the bronchus sign in computer tomography (CT) has been used to determine if the lesion is reachable by R-EBUS [4], several lesions cannot be reached even when the bronchus sign is prominent. Geometric obstacles such as the tortuosity of the bronchus and the acute angle between the bronchi are possible candidates for preventing access to the lesion. Due to the high inter-observer variability and difficulty in measuring these parameters, a study using quantitative CT scan imaging is required.

Grélard et al. conducted a geometric analysis of tubular organs in medical images, including centerline extraction, shape analysis, and topological analysis [5]. These methods have been applied in the analysis of coronary arteries and brain vessels and have shown promise in understanding the structural characteristics of these tubular structures [6]. The Vascular Modeling Toolkit (VMTK) [7] is a popular open-source tool for modeling the structure of vascular structures. The CRIMSON method [8] uses VMTK as an element of cardiovascular modeling. Given that the airway system is also a tubular structure, the application of these open-source tools could be considered after proper modification and optimization to analyze the specific structure and geometry of the airway.

Consequently, this study aimed to quantitatively reveal contributing factors to airway navigation failure during R-EBUS by using geometric analysis in a 3D space and to investigate the clinical feasibility of prediction models for airway navigation failure.

## Methods

**Fig. 1:**
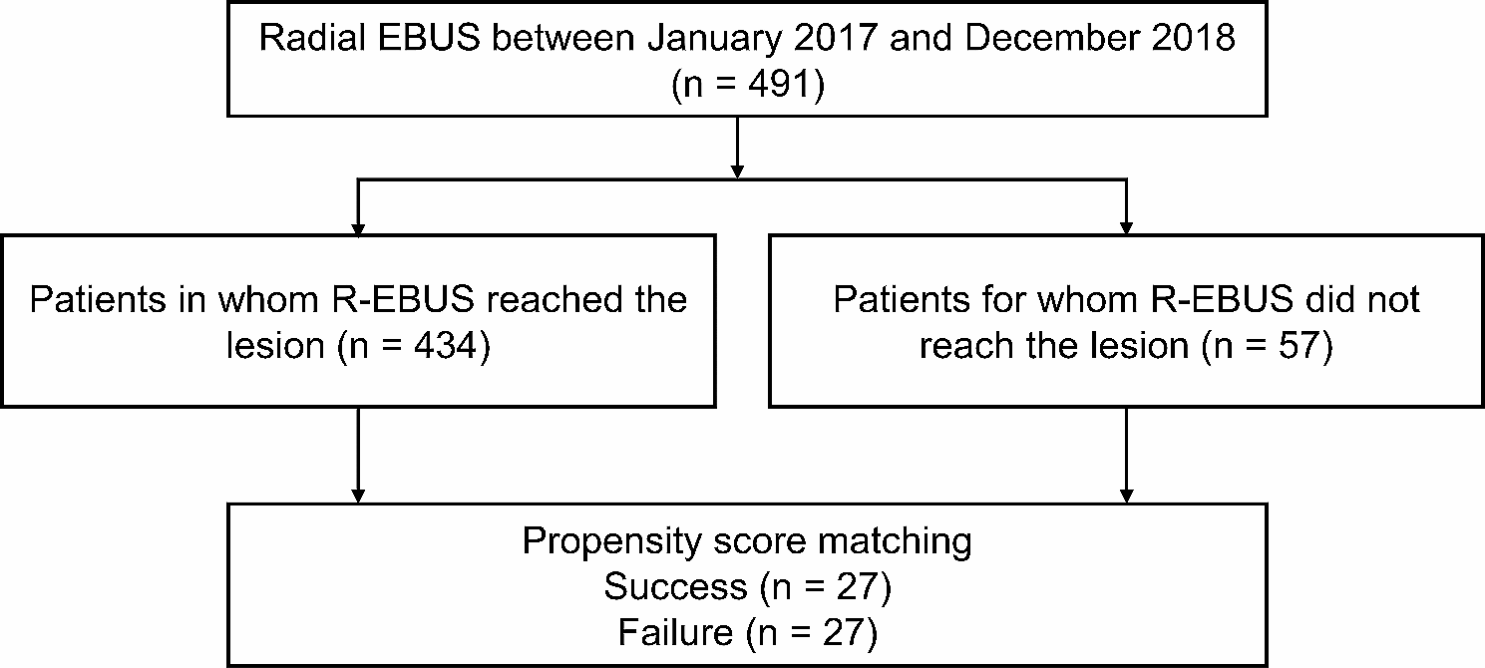
Flow chart of patient

This study was approved by the Samsung Medical Center Institutional Review Board (SMC IRB no. 2018-03-021). Informed consent was waived because of the observational nature of the study. We retrospectively reviewed all patients who underwent R-EBUS between January 2017 and December 2018 at a tertiary referral hospital with 2000 beds in Seoul, Korea. Patients in whom R-EBUS reached the lesion were classified as the success group, whereas patients who had a leading bronchus to the target lesion that was not reached by R-EBUS were classified as the failure group. Twenty-seven patients in the failure group were matched with 27 patients in the success group based on propensity scores (Fig. 1).

CT images were obtained with the following parameters: detector collimation, 1.25–0.625 mm; 120 kVp; 150–200 mA; and a reconstruction interval of 1–2 mm. R-EBUS-guided transbronchial lung biopsy was performed under conscious sedation induced with midazolam and fentanyl to evaluate the tracheobronchial tree. A 4.1-mm bronchoscope (BF P260F; Olympus, Tokyo, Japan) was used to reach the sub-subsegmental level nearest to the lesion after reviewing CT. Then, the R-EBUS probe (1.4-mm, 20-MHz, UM S20-17 S; Olympus) was inserted through the bronchoscope working channel. Reaching the target lesion for endobronchial navigation was considered a success. When the nodule was invisible to the radial probe, it was defined as a failure of endobronchial navigation using thin bronchoscopy.

**Fig. 2:**
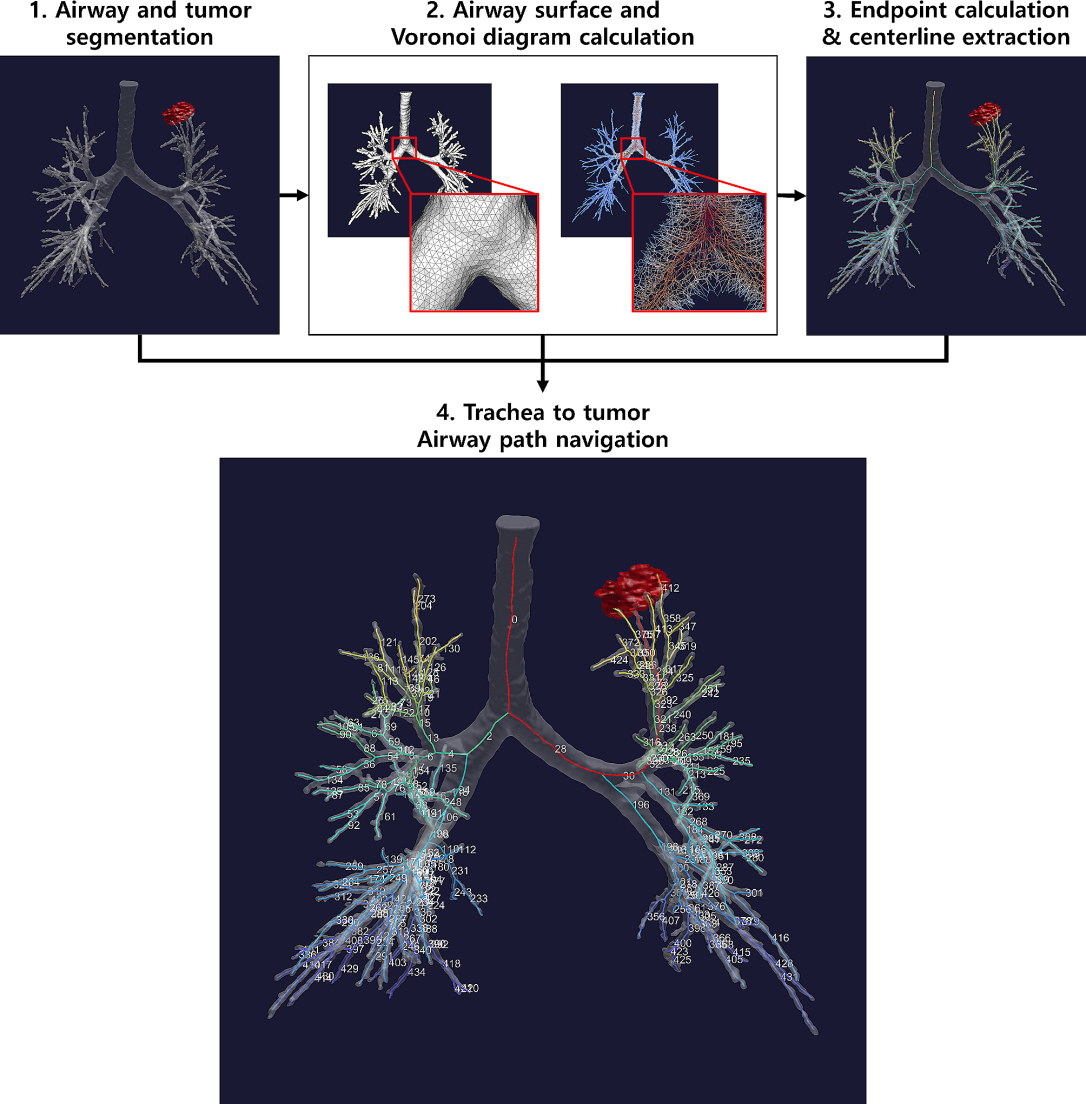
Overall workflow of the study

We built the lung geometric characteristic quantification pipeline based on the segmented airway mask from CT images. Fig. 2 shows the overall workflow of our study. We first applied a region-growing algorithm on a CT image to extract a candidate airway mask [9]. The resulting mask was then used as input for further processing steps, including centerline extraction, shape analysis, and geometric analysis. To calculate the surface nodes and edges of the binary mask, a marching cube algorithm was applied and spatial interpolation was used to adjust the coordinates of each node to create isotropic triangles [10, 11]. A 3D Voronoi diagram was then calculated to evenly divide the volume space between each surface node, and the edges of each Voronoi plane were used as centerline candidates [5]. Centerline tracking was subsequently performed along the edges of the Voronoi diagram, starting from the top of the trachea and ending at the termination of each airway. The starting and endpoints for this process were determined using 3D skeletonization [12] applied to the binary airway mask, resulting in a tree structure with leaf nodes defined as endpoints and the first node defined as the starting point automatically.

To quantify the geometric characteristics of the airway, we calculated various features at every centerline point. These included the area, perimeter, minimum diameter, maximum diameter, and circularity of the airway cross-sectional plane perpendicular to the centerline direction. We also calculated the curvature and torsion of the centerline, as well as the diameter of the maximum inscribed sphere at each point using the centerline and airway surfaces. Additionally, we divided the airway into sections according to the bifurcation. Then we calculated the length of each section and the bifurcation angle at bifurcation points.

The processes were implemented using open-source python libraries including the Vascular Modeling Toolkit (VMTK, http://www.vmtk.org) [7], simple insight toolkit (https://sitk.org) [13, 14], and sci-kit image (https://scikit-image.org) [15]. We developed a wrapper code with a graphical user interface using pyQt5, allowing for easy use by medical staff (https://github.com/Hwan-ho/AirGeo).

To assess the effectiveness of the significant features identified in our analysis, we trained three distinct machine learning models using these features: a support vector machine (SVM), a random forest (RF) model consisting of 512 decision trees, and an L1-norm regularized logistic model. To prevent overfitting and evaluate performance, 1000 times bootstrapping was used with a 0.3 hold-out ratio. We trained the models on the training set during each iteration and evaluated their performance on the test set. We assessed the performance of each model using various metrics, including accuracy, sensitivity, specificity, and area under the receiver operating characteristic curve. Additionally, we analyzed the relative importance of each feature using the selection frequency of a Logistic Lasso model to determine which features were most predictive of failure.

To minimize the differences between the baseline characteristics of the groups, propensity score matching was conducted. A propensity score was calculated using a non-parsimonious logistic regression model that included gender, lobe, and order of leading bronchus to the lesion. Propensity score matching was performed using 1:1 matching and a caliper of 0.25. When the standardized mean difference between selected variables was less than 0.2, we deemed a balance to have been achieved. To compare continuous clinical variables, we used two-sample Student’s t-tests, and for discrete clinical variables, we used chi-square tests. The mean and standard deviation were reported for continuous variables. A *P*-value less than 0.05 was considered statistically significant. All statistical analyses were performed using the Statistics and Machine Learning Toolbox in MATLAB (The MathWorks, Natick, MA).

## Results

Of the 491 patients who were eligible for analysis, the target lesion was reached in 434 and was not reached in 57. The baseline characteristics of both groups are presented in Table 1. Before propensity score matching, we observed that the median of the orders of Branch0 was significantly lower in the success group than in the failure group (3.9 *versus* 5.7, *P < .*001). Gender, FEV1, and the location of the target lesion were not different between the two groups. After matching, 27 patients were finally involved for each group, and non-significant statistical differences in characteristics were observed between the groups. Fig. S1 shows the t-SNE embedding results of the clinical parameters before and after propensity matching [16].

**Table 1:** Clinical characteristics in the study population

Variables	Total patients				Propensity-matched patients			
	Success (*n =* 434)	Failure (*n* = 57)	*P* Value	SMD	Success (*n =* 27)	Failure (*n =* 27)	*P* Value	SMD
Age (years)	65 ± 11 66 (58–73)	63 ± 13 64 (57–72)	0.285	0.141	64 ± 7 63 (60–67)	64 ± 15 69 (50–77)	0.860	0.049
Male, n (%)	240 (55)	34 (60)	0.534	0.088	19 (70)	17 (63)	0.564	
FEV1	88 ± 15 88 (79–98)	85 ± 23 88 (72–103)	0.473	0.123	88 ± 12 92 (80–96)	88 ± 20 96 (73–104)	0.927	0.028
Location			0.815				0.889	
Upper lobe	215 (50)	30 (53)		0.062	15 (56)	13 (48)		0.148
Lingular division or middle lobe	58 (13)	6 (11)		0.088	1 (4)	1 (4)		0
Lower lobe	161 (37)	21 (37)		0.005	11 (41)	13 (48)		0.149
Order of Branch0	3.9 ± 0.9 4.0 (3.0–4.0)	5.7 ± 0.7 6.0 (5.0–6.0)	< 0.001	2.113	5.6 ± 0.8 5.0 (5.0–6.0)	5.6 ± 0.7 5.0 (5.0–6.0)	0.891	0
Long axis diameter (mm)	30 ± 13 28 (22–36)	28 ± 11 26 (21–34)	0.779	0.1884	22 ± 8 22 (16–25)	30 ± 13 27 (21– 39)	0.010	0.805

Note.—Data are reported as mean ± standard deviation, median (interquartile range), and numbers (%)

FEV1 = forced expiratory volume in 1 s, SMD = standardized mean difference

A comprehensive list of the computed geometric features can be found in Table 2 and the graphical description is shown in Fig. S2. To identify differences between successful and failed cases, we evaluated first order statistics including the minimum, maximum, and average features of each airway section. Furthermore, we calculated the total path length from the top of the trachea to the lesion, the summation of bifurcation angles in the path, and the minimum, maximum, average, and standard deviation of bifurcation angles in the path. We also calculated the sectional length of each branch and the bifurcation angle to enter each branch. As a result, 38 features per branch section and six global scale features are used to assess the failure factor. We assigned a unique name to each feature by combining its feature name with its corresponding branch number. The closest branch to the lesion is designated as Branch 0, with the branch number increasing as the distance from Branch 0 increases. For instance, the branch section number of the branch located immediately before the closest branch to the lesion is referred to as Branch 1.

**Table 2:** List of airway geometry features

Scale	Feature	Statistics
Global		
	Total length	-
	Bifurcation angles in the path	Sum, minimum, maximum, average, standard deviation
Local		(Sectional statistics)
	Bifurcation angle for entrance	-
	Sectional length	-
	Cross-sectional area	Minimum, Maximum, average, standard deviation
	Maximum inscribed sphere radius	Minimum, Maximum, average, standard deviation
	Minimum diameter	Minimum, Maximum, average, standard deviation
	Maximum diameter	Minimum, Maximum, average, standard deviation
	Min-Max diameter ratio	Minimum, Maximum, average, standard deviation
	Curvature	Minimum, Maximum, average, standard deviation
	Torsion	Minimum, Maximum, average, standard deviation
	Perimeter	Minimum, Maximum, average, standard deviation
	Circularity	Minimum, Maximum, average, standard deviation

**Fig. 3:**
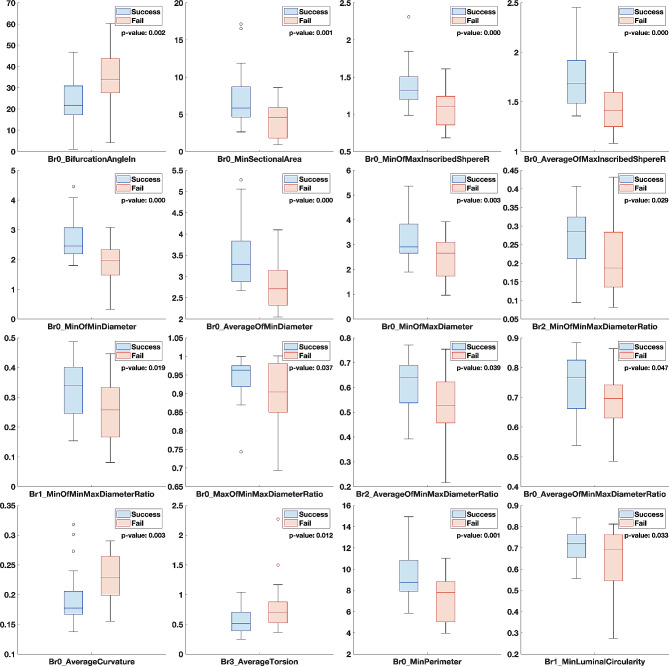
Airway geometry features that exhibited significant differences between the success and failure groups

Figure 3; Table 3 show the features that show a significant difference between success and failure cases. The results of our analysis revealed that 16 features exhibited significant differences between the success and failure groups. The majority of significant features were found in the last branch (Branch0) and were mainly related to its thickness, such as the minimum SectionalArea, minimum MaxInscribedSphereR, average MaxInscribedSphereR, minimum MinDiameter, average MinDiameter, minimum MaxDiameter, and minimum Perimeter. According to the box chart in Fig. 3, the last branch of failure cases was considerably narrower than that of successful cases. The next significant features were related to the possibility of a bend occurring in Branch0 while navigating, including BifurcationAngleIn and the average of Curvature. Furthermore, the cross-sectional shape of branches (i.e., MinMaxDiameterRatio and LuminalCircularity) were also significant features. We conducted feature selection using the Logistic Lasso model during 1000-times bootstrapping and calculated the selection probability and their coefficient (Table S1).

**Table 3:** Significantly different features between success and failure group

Branch No.	Feature names	Statistics	Success	Failure	*P* Value
0	BifurcationAngleIn	-	23.174 (11.243)	34.256 (12.971)	0.002
0	SectionalArea	Minimum	7.139 (3.721)	4.170 (2.384)	0.001
0	MaxInscribedSphereR	Minimum	1.384 (0.294)	1.089 (0.261)	< 0.001
0	MaxInscribedSphereR	Average	1.744 (0.282)	1.438 (0.231)	< 0.001
0	MinDiameter	Minimum	2.670 (0.672)	1.946 (0.643)	< 0.001
0	MinDiameter	Average	3.547 (0.753)	2.782 (0.525)	< 0.001
0	MaxDiameter	Minimum	3.182 (0.836)	2.467 (0.820)	0.003
2	MinMaxDiameterRatio	Minimum	0.269 (0.084)	0.212 (0.101)	0.029
1	MinMaxDiameterRatio	Minimum	0.323 (0.093)	0.259 (0.103)	0.019
0	MinMaxDiameterRatio	Maximum	0.942 (0.055)	0.901 (0.086)	0.037
2	MinMaxDiameterRatio	Average	0.607 (0.111)	0.539 (0.128)	0.039
0	MinMaxDiameterRatio	Average	0.737 (0.102)	0.684 (0.091)	0.047
0	Curvature	Average	0.193 (0.045)	0.230 (0.041)	0.003
3	Torsion	Average	0.552 (0.195)	0.792 (0.423)	0.012
0	Perimeter	Minimum	9.430 (2.318)	7.236 (2.174)	0.001
1	LuminalCircularity	Minimum	0.709 (0.082)	0.635 (0.156)	0.033

Note.—Units: BifurcationAngleIn (degree); SectionalArea (mm^2^); MaxInscribedSphereR, MinDiameter, MaxDiameter, Perimeter (mm); Curvature (1/mm); Otherwise (None)

**Fig. 4:**
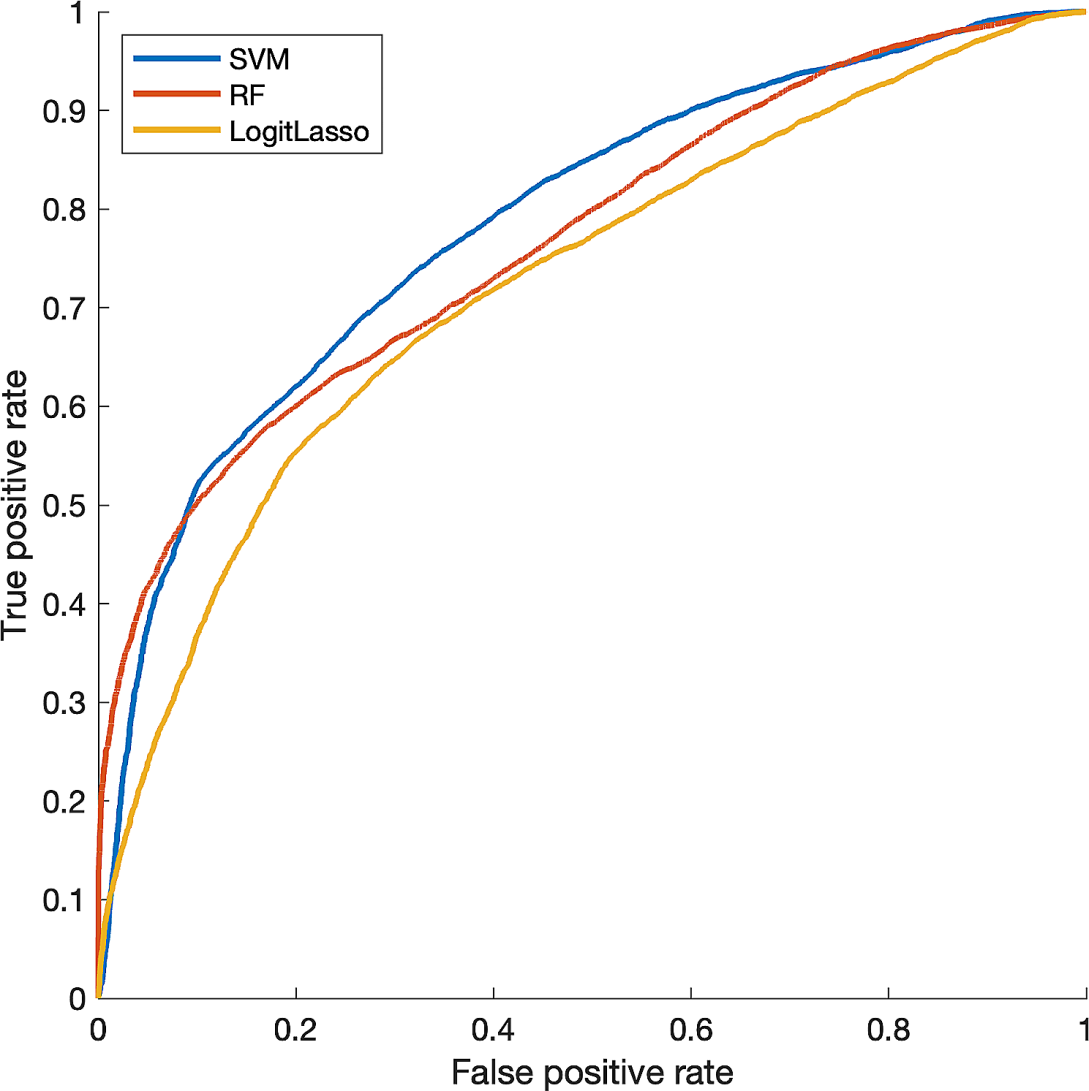
Averaged receiver operating characteristic curve of each classifier in the test phase The area under the curve was 0.803, 0.781, and 0.750 for SVM, RF, and Logistic Lasso classifiers, respectively

Table 4 presents the results of using machine learning to predict navigation failure based on significant features. Each performance measure is the average and standard deviation of 1000 bootstrapped samples using a hold-out ratio of 0.3. In the confusion matrix, positive cases were defined as failures. The average area under the curve (AUC) of receiver operating characteristic curve in the test phase was 0.803, 0.781, and 0.750 for SVM, RF, and Logistic Lasso models, respectively (Fig. 4). The AUC of the SVM model was statistically significantly higher than the other models (*P*-value < 0.001 for both). Therefore, using an SVM is the most effective method for predicting airway navigation failure.

**Table 4:** Evaluation of airway navigation failure prediction performance through 1,000 bootstrapped samples

	Training			
	Accuracy	Sensitivity	Specificity	AUC
SVM	0.776 (0.039)	0.620 (0.07)	0.932 (0.037)	0.880 (0.031)
RF	1.000 (0.000)	1.000 (0.000)	1.000 (0.000)	1.000 (0.000)
Logistic Lasso	0.813 (0.065)	0.790 (0.065)	0.836 (0.114)	0.908 (0.061)

	Test			
	Accuracy	Sensitivity	Specificity	AUC
SVM	0.711 (0.091)	0.528 (0.169)	0.895 (0.124)	0.803 (0.096)
RF	0.687 (0.096)	0.648 (0.159)	0.725 (0.155)	0.781 (0.092)
Logistic Lasso	0.673 (0.096)	0.664 (0.151)	0.681 (0.170)	0.750 (0.098)

Note.—The number enclosed in parentheses denotes the standard deviation

SVM = support vector machine; RF = random forest; AUC = area under the curve

**Fig. 5:**
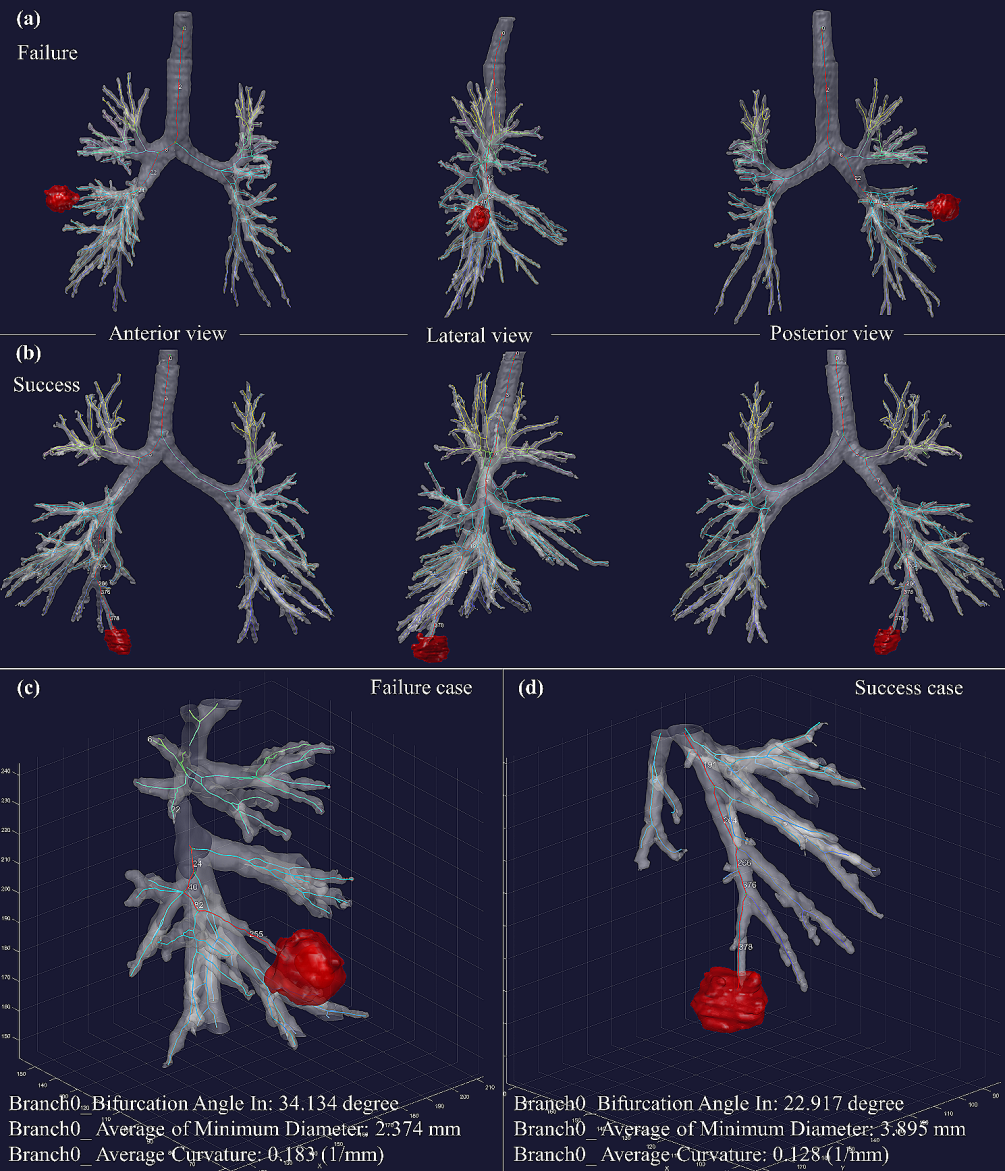
Comparison of representative cases. (**a**) Anterior, lateral, and posterior views of a failure case where the lesion is located in the superior segment of the right lower lobe, (**b**) Anterior, lateral, and posterior views of a success case where the lesion is located in the posterior segment of the right lower lobe. (**c**) Enlarged 10 mm × 10 mm × 10 mm region around tumor of failure case. (**d**) Enlarged 10 mm × 10 mm × 10 mm region around tumor of success case. The white numbers correspond to the unique index of each branch section

Figure 5 depicts a representative case of both success and failure. Although the naked eye can identify slight differences, the quantitative value calculated from airway geometry shows a difference. The failure case exhibits a larger bifurcation angle to enter Branch0, which is the nearest branch to the lesion (i.e., branch index 255 for the failure case and branch index 378 for the success case). Moreover, Branch0 of the failure case has a narrower diameter and a higher curvature than the success case. These results demonstrate the feasibility of the pipeline to capture geometric differences between success and failure cases during airway navigation.

## Discussion

This study aimed to quantitatively reveal contributing factors to airway navigation failure during R-EBUS by using geometric analysis in a 3D space and to investigate the clinical feasibility of prediction models for airway navigation failure. We analyzed the 3D geometric features of the airway from the top of the trachea to the lesion and discovered that a large bifurcation angle and a narrow and tortuous structure of the closest bronchus from the lesion are significant factors in airway navigation failure. Moreover, we used a machine learning-based approach to explore the utility of these significant factors. The SVM model can predict airway navigation failure with an average AUC of 0.803.

In recent years, there have been notable advancements in image-guided bronchoscopy modalities for peripheral pulmonary lesions. Although several techniques are available, their low diagnostic performance needs further improvement. In a recent meta-analysis, the pooled sensitivity of RP-EBUS was 0.72 (95% CI, 0.70–0.75) [2]. A meta-analysis that included 11 studies for electromagnetic navigation bronchoscopy reported a diagnostic yield of 67% [17]. Robotic bronchoscopy, the newest and most expensive modality, has been reported to have a diagnostic yield of only 74%, despite reaching the lesion in 96% of cases [18]. Bronchus signs are widely used but have high inter-observer variability and need to be combined with other factors to predict airway navigation failure [19]. Our study is the first to quantitatively analyze the geometric obstacles that prevent a radial probe from reaching the target lesion.

The BifurcationAngleIn, average MinDiameter, and average Curvature at Branch 0 were the most frequently selected features. The BifurcationAngleIn of Branch 0 has a positive coefficient, indicating that higher bifurcation angles at the last branch section tend to cause airway navigation failure. The sectional average MinDiameter in Branch 0 has a negative coefficient, indicating that a narrow diameter of the last airway branch lumen can cause failure. The sectional average of Curvature in Branch 0 has a positive coefficient, indicating that the presence of highly tortuous structures at the end of the airway section can likely cause airway navigation failure. In summary, the bifurcation angle at the target branch, the average diameter of the last section, and the curvature of the last section are the most significant and stable factors for airway navigation failure. The end of the airway in the failed case appears to be narrower and more twisted. These geometric obstacles are thought to prevent the advance of the R-EBUS probe.

The factors that predict airway navigation failure are poorly understood. Three retrospective studies reported that small lesion sizes (< 20 mm) predicted airway navigation failure [3, 20, 21]. However, in our study, among all patients, the size of the failure group was a median of 26 mm, which was not significantly different from the size of the success group, which was 28 mm. This indicates that understanding the geometry of the airway to the lesion is more important for diagnostic success than size. In one of these studies, lesions close to the pleura (< 10 mm) were significantly associated with airway navigation failure [20]. Tay et al. reported a distance of 40 mm or less from the hilum as a significant factor in reaching the lesion [21]. In contrast, the total distance to the lesion was not significantly associated with airway navigation failure in our study. We believe this because, after propensity score matching, the order of the leading bronchus to the lesion was similar in both groups. Imabayashi et al. recently reported that subdividing the bronchus sign offers a more accurate prediction of reaching the target lesion [4]. We expect that subtyping the bronchus sign with quantitative CT analysis will be a good predictor. Based on this study, we plan to develop a more accurate prediction model by adding clinical variables such as lesion size and quantified bronchus-lesion relationships.

Quantitative CT scan imaging is increasingly applied to quantify the mucus plug, emphysema, and airway measurement in various lung diseases, including interstitial lung disease [22], chronic obstructive pulmonary disease [23], cystic fibrosis [24], asthma [25], and bronchiectasis [26]. These quantifications in small airway disease provide us with insights into the pathogenesis [27], clinical presentation [28], diagnosis [29], and prognosis [30–32]. Despite advances in quantitative analytics, few quantitative CT scan measurements are applied in real-world clinical practice to guide treatment decisions or to judge treatment outcomes [33]. Our study suggests that quantitative CT scan imaging has the potential to improve the yield of diagnostic procedures. Accurate predictive models might reduce the cost and complications of unnecessary procedures, while also helping non-experts to recognize lesions that could be easily diagnosed.

This study has several limitations. First, this was a retrospective, small study conducted at a single hospital. Although a propensity score analysis was used, selection bias may have affected the results. Second, the results of geometric feature calculation are largely dependent on the accuracy of airway mask segmentation. In this study, we attempted to refine the airway mask using image processing techniques such as surface smoothing. However, to ensure reliable geometric feature calculations, it is crucial to conduct a robust airway mask segmentation process. Third, the lack of use of familiar features such as size or subclassification of bronchus sign in the prediction model could be challenging for clinicians to accept. However, we focused on whether quantitative analysis of airway, which was not previously available to clinicians, could help predict diagnostic success. We also believe that quantitative analysis of entry pathways into the lesion will ultimately include the influence of conventional clinical variables, including size and bronchus signs. Fourth, because the R-EBUS was performed by a highly experienced bronchoscopist, the results cannot be generalized.

## Conclusions

Geometric analysis in 3D space revealed that a large bifurcation angle and a narrow and tortuous structure of the closest bronchus from the lesion are associated with airway navigation failure during R-EBUS. Predictive models developed using quantitative CT scan imaging have the potential to improve the yield of diagnostic procedures.

### Electronic supplementary material

Below is the link to the electronic supplementary material.

Supplementary Material 1

## Data Availability

All data generated or analyzed during the study are included in the published paper.

## Abbreviations

AUC: Area under the curve
CT: Computed Tomography
FEV1: Forced Expiratory Volume in 1 s
R-EBUS: Radial Endobronchial Ultrasound
RF: Random Forest
SVM: Support Vector Machine
VMTK: Vascular Modeling Toolkit
