# Supplementary material for: 3D airway geometry analysis of factors in airway navigation failure for lung nodules
Source: Cancer Imaging. 2024 Jul 4;24:84. doi: 10.1186/s40644-024-00730-7 (PMC11223435; doi:10.1186/s40644-024-00730-7)
Supplement: Supplementary file 1 — Supplementary Material 1 [file 40644_2024_730_MOESM1_ESM.docx]

**Table S1: Selection Probability and Coefficients of Significant Features**

| Branch  No. | Feature names | Statistics | Selection  probability | Coefficient (95%CI) | *P* Value |
| --- | --- | --- | --- | --- | --- |
| **0** | ***BifurcationAngleIn*** | ***-*** | **0.668** | 0.015 (0.014, 0.016) | <.001 |
| 0 | SectionalArea | Minimum | 0.052 | -0.004 (-0.005, -0.002) | <.001 |
| 0 | MaxInscribedSphereR | Minimum | 0.305 | -0.289 (-0.33, -0.248) | <.001 |
| 0 | MaxInscribedSphereR | Average | 0.238 | -0.32 (-0.369, -0.272) | <.001 |
| 0 | MinDiameter | Minimum | 0.509 | -0.266 (-0.29, -0.242) | <.001 |
| **0** | ***MinDiameter*** | ***Average*** | **0.787** | -0.497 (-0.524, -0.469) | <.001 |
| 0 | MaxDiameter | Minimum | 0.086 | -0.032 (-0.04, -0.023) | <.001 |
| 2 | MinMaxDiameterRatio | Minimum | 0.601 | -1.996 (-2.148, -1.844) | <.001 |
| 1 | MinMaxDiameterRatio | Minimum | 0.274 | -0.628 (-0.727, -0.53) | <.001 |
| 0 | MinMaxDiameterRatio | Maximum | 0.165 | -0.379 (-0.474, -0.284) | <.001 |
| 2 | MinMaxDiameterRatio | Average | 0.333 | -0.453 (-0.514, -0.392) | <.001 |
| 0 | MinMaxDiameterRatio | Average | 0.586 | -2.262 (-2.439, -2.084) | <.001 |
| **0** | ***Curvature*** | ***Average*** | **0.718** | 5.900 (5.490, 6.310) | <.001 |
| 3 | Torsion | Average | 0.636 | 0.452 (0.412, 0.492) | <.001 |
| 0 | Perimeter | Minimum | 0.063 | -0.006 (-0.008, -0.004) | <.001 |
| 1 | LuminalCircularity | Minimum | 0.366 | -0.706 (-0.795, -0.616) | <.001 |

CI = confidence interval.

**Figure S1:** t-SNE embedding result of clinical parameters before and after the propensity matching. The discrepancy between the success and failure groups diminished after performing propensity matching.


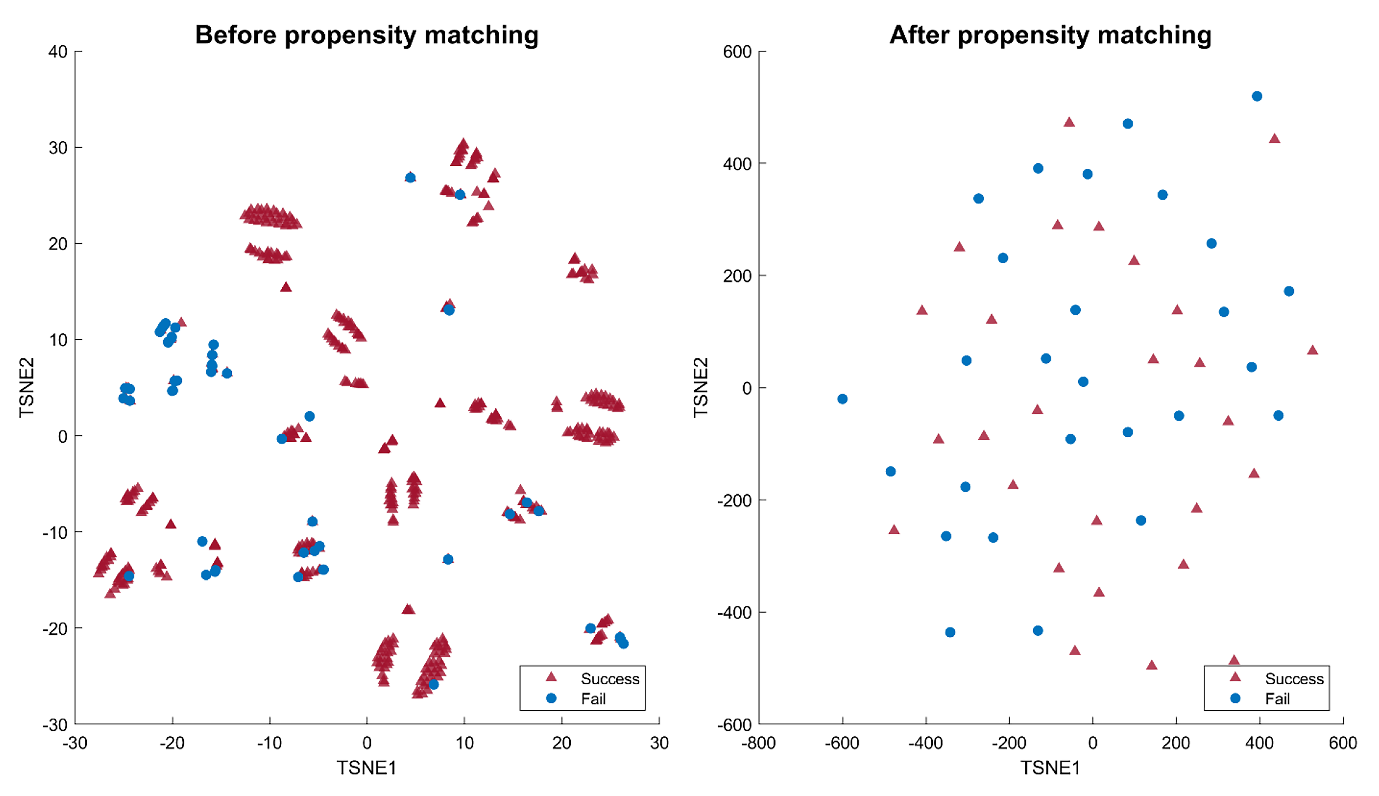


**Figure S2:** Graphical description of airway features

(**a**) Minimum and maximum diameter, (**b**) curvature, (**c**) sectional area, (**d**) maximum inscribed sphere, and (**e**) bifurcation angle in

**
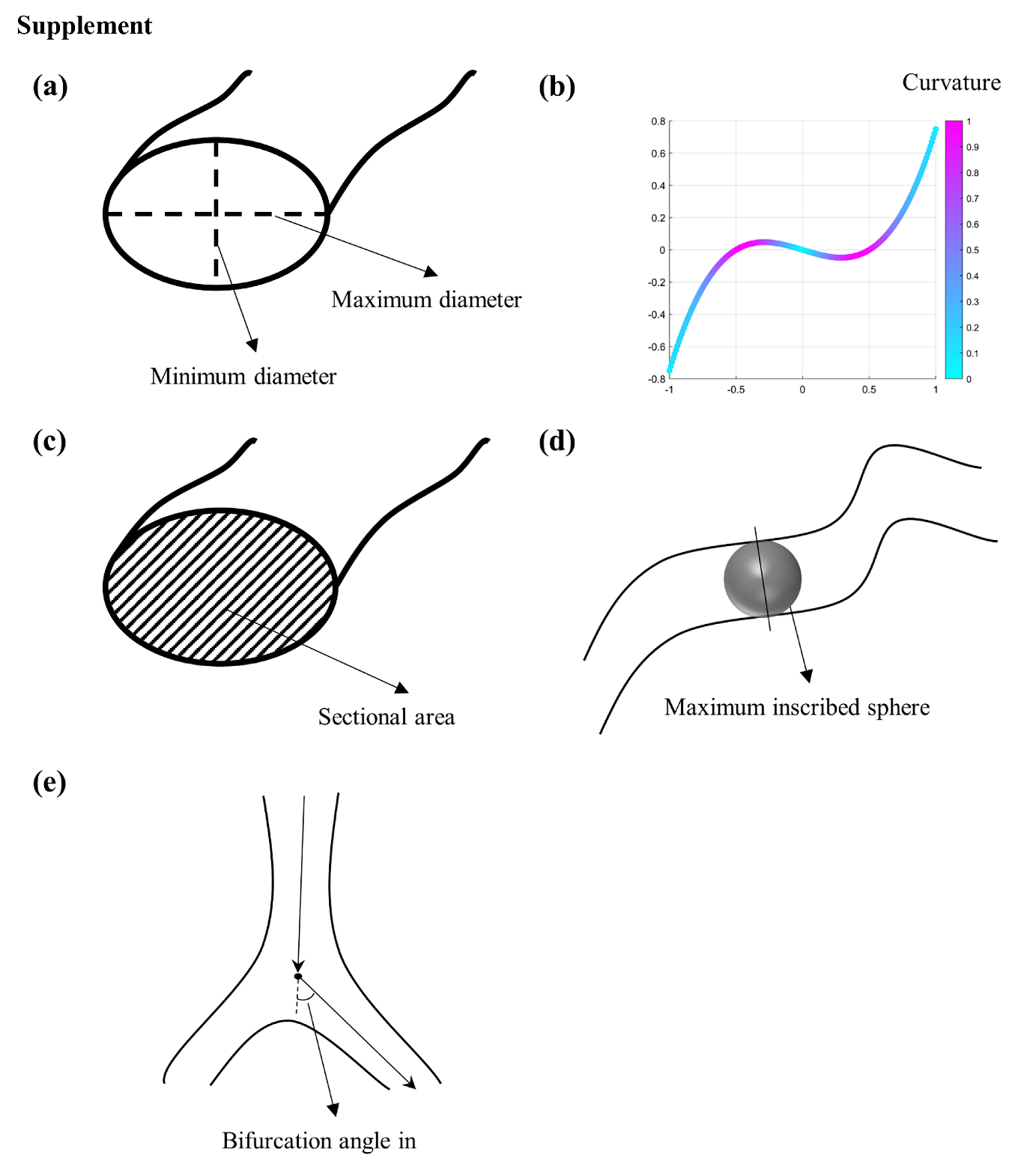
**
